# What Are the Sensory Attributes Associated with Consumer Acceptance of Yellow Oyster Mushrooms (*Pleurotus citrinopileatus*)?

**DOI:** 10.3390/foods13132061

**Published:** 2024-06-28

**Authors:** Minji Oh, Jin-Hee Ju, Seyoung Ju

**Affiliations:** 1 Mushroom Research Division, National Institute of Horticultural & Herbal Science, RDA, Eumseong 27709, Chungbuk, Republic of Korea; minji1228@korea.kr; 2 Department of Green Convergence Technology, College of Science and Technology, Konkuk University, Chungju-si 27478, Chungbuk, Republic of Korea; jjhkkc@kku.ac.kr; 3 Department of Food & Nutrition, College of Biomedical and Health Science, Konkuk University, Chungju-si 27478, Chungbuk, Republic of Korea

**Keywords:** yellow oyster mushroom, descriptive analysis, consumer acceptability, partial least square regression (PLSR) analysis

## Abstract

The oyster mushroom is cultivated globally, renowned for its unique texture and umami flavor, as well as its rich content of nutrients and functional ingredients. This study aims to identify the descriptive sensory characteristics, assess the consumer acceptability of new superior lines and cultivars of yellow oyster mushrooms, in addition to exploring the relationship between these descriptive characteristics and consumer acceptability. Statistical analyses were performed using one-way analysis of variance (ANOVA), principal component analysis (PCA), and partial least squares regression (PLSR). Twenty attributes were delineated, including three related to appearance/color (gray, yellow, and white), four associated with the smell/odor of fresh mushroom (oyster mushroom, woody, fishy, and seafood smells), three pertaining to the smell/odor of cooked mushrooms (mushroom, umami, and savory smells), four describing flavor/taste (sweet, salty, umami, and savory tastes), and five for texture/mouthfeel (chewy, smooth, hard, squishy, and slippery textures). Consumer acceptability tests involved 100 consumers who evaluated overall liking, appearance, overall taste, sweetness, texture, savory taste, MSG taste, smell, color, purchase intention, and recommendation. The general oyster mushroom (548 samples) scored highest in acceptability. Seven attributes, namely fresh mushroom smell, seafood smell (fresh), fishy smell (fresh), umami smell (cooked), nutty smell (cooked), salty taste, and MSG taste with the exception of appearance showed significant differences among samples (*p* < 0.001). The three yellow oyster mushroom samples were strongly associated with attributes like hardness, softness (texture), sweet taste (745 samples), MSG taste, salty taste, squishy texture, and fishy smell (483 and 629 samples). The development of sensory lexicons and increasing consumer acceptance of new superior lines and cultivars of yellow oyster mushroom will likely enhance sensory quality and expand the consumer market, aligning with consumer needs and preferences.

## 1. Introduction

Typical mushrooms are fleshy fruiting bodies of fungi that belong to the family Agaricale, and edible mushrooms can be harvested from nature or cultivated [1,2]. There are over 2000 species of edible mushrooms, yet only about 35 species are commercially cultivated [1]. Besides their unique texture and flavor are highly valued for their rich content of nutrients and functional ingredients.

The genus *Pleurotus*, which includes oyster mushrooms, is extensively cultivated alongside the button and shiitake mushrooms due to its ability naturally thrive in most regions of the world, comprising 16% of the total global edible mushroom production [3,4]. In Korea, oyster mushrooms are the most widely cultivated and consumed. The species of oyster mushrooms cultivated globally include *Pleurotus ostreatus* (oyster mushrooms), *P. florida* (Florida oysters), *P. pulmonarius* (Lung oysters), *P. eryngii* (King oysters), *P. citrinopileatus* (yellow oysters), *P. salmoneostramineus* (pink oysters), and *P. cystidiosus* (abalone oysters). Yellow oyster mushrooms, predominantly found in Korea, Japan, northeastern China, Russia, Europe, and North America, are commercially cultivated mainly in Korea, Japan, and Russia. Yellow oyster mushrooms are a robust source of nutrients such as protein, dietary fiber, vitamins, and minerals, as well as bioactive compounds including α-glucans, β-glucans, lentanin, lipopolysaccharides, and resveratrol [5]. These nutritive elements have been linked nutraceutical and therapeutic activities such as antioxidant, anti-inflammatory, anti-tumor, antibacterial, and hypocholesterolemic effects [6,7,8,9,10]. Owing to these nutritional and therapeutic benefits, mushroom research has expanded significantly, encompassing nutritional and bioactive studies as well as the processing of techniques [11,12,13]. Mushrooms are also prized for their distinctive texture and umami flavor from culinary science perspective [11]. However, the distinctive texture and taste of mushrooms significantly influence consumer preferences, notwithstanding their health benefits. The importance of research on the sensory properties of mushrooms is growing, yet studies involving trained panels or consumers to evaluate these properties are scant [14,15,16,17]. Thus, focusing on sensory property research is crucial for innovating new mushroom products in the global market. Moreover, the unique texture and functional compounds of mushrooms are emerging as vital ingredients in future foods, such as alternatives to meat or other plant-based products, in response to increasing demands for healthier and more sustainable foods.

This study aims to identify the sensory descriptors and examine consumer acceptance of new superior lines and cultivars of yellow oyster mushroom, as well as to explore the relationship between sensory attributes and consumer acceptability of new superior lines. The findings will contribute essential data for the development of new cultivars aimed at enhancing flavor, texture and production levels of yellow oyster mushrooms. It is anticipated that this research will facilitate the development of new products that are beneficial to health and preferred by consumers.

## 2. Materials and Methods

### 2.1. Samples

**Figure 1:**
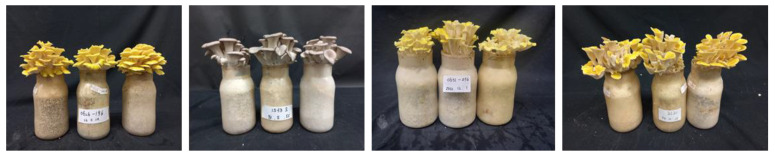
Morphologies of fruiting body of oyster mushrooms from left Pc-21-ja196, Otari, Pc-21-ba276, and Jangdari.

Table 1 presents details on mushroom strains, including information about each sample, and their respective random sample numbers, while Figure 1 illustrated the morphologies of the fruiting bodies of oyster mushrooms. The mushroom samples were harvested by the Mushroom Research Division of the National Institute of Horticultural and Herbal Science in January. The sample labeled 548 is the -“Otari”- cultivar of oyster mushroom (KMCC5275, *Pleurotus ostreatus*), developed by the Mushroom Research Division of the National Institute of Horticultural and Herbal Science in 2021. Conversely, 745 corresponds to the – “Jangdari”- cultivar of yellow oyster mushroom (KMCC0422, *Pleurotus citrinopileatus*), which was also developed by this division in 2012 [18]. Sample 483 was obtained through crossbreeding two monokaryons from KMCC0422 and KMCC2159 in 2021 and was selected in 2023, This newly developed yellow oyster mushroom line is characterized by its high productivity and ability to thrive at lower temperatures. Similarly, sample 629 emerged from the crossbreeding of monokaryons from KMCC0429 and KMCC2159 in 2021 and was selected in 2023, showing similarly high productivity. These samples were freshly prepared and cooked to evaluate their aroma. Specifically, the samples were steamed for five minutes to assess cooked mushrooms, whereas the fresh samples were washed in running water prior to evaluation. Subsequently, the samples measured at 10 g each (comprising 3 to 4 pieces), were placed in white paper cups measuring 7 cm in diameter and 6 cm in height and were presented to panelists. And water also were presented panelists to rinse their mouths between each sample. The samples were coded with three-digit random numbers and presented using a Latin square design to minimize carry-over effects [19].

### 2.2. Panelists

Panel candidates, experienced in food sensory evaluation, were recruited from the local community. After taste sensitivity and discrimination of tastes, ten panelists (five females and five males, aged 20~40) were selected. They completed general sensory training which enhanced their skills articulating and evaluating sensory characteristics. During the initial 10-hour session, the panelists were trained on basic taste test, as well as recognitions of flavor and aroma, they also learned descriptive scaling methods for appearance, smell/aroma, flavor, and texture, including standard references, and intensity ranking tests for sensory evaluation descriptors [20]. This training continued over five days, lasting two hours each day. Subsequently, in the 60-hour second training session. Panelists were instructed in descriptive sensory techniques, focusing on attribute identification and intensity rating, two hours daily for three months. The study received approval from the Konkuk University Institutional Review Board (7001355-202402-HR-758).

### 2.3. Sensory Evaluation Procedure

In the initial 2-h session, the panelists evaluated four mushroom samples, describing each sample attributes of mushrooms for appearance, odor/smell, flavor/taste, and texture of samples. An open discussion led by a panel leader followed, to achieve consensus on the attributes and establish terminology and definitions. In the subsequent 2-h session, the panelists refined their set of descriptive attributes by evaluating various, continuously tasting, discussing, and reaching agreement among all panelists.

For the third session (10 h), the lexicon was clarified initially. Following this, a range of materials were presented to the panelists for the selection of standard references, which took four hours. The standard references were chosen through panel discussions, guided by the panel leader. During the development of the lexicon and the selection of standard references, our focus was on savory, meat-like, or umami flavor and tastes. Reference samples, comprising ingredients known for their umami taste such as beef broth and broth from fish and shellfish were prepared; subsequently, the standard references were selected following consensus among the panelists. The Descriptive attributes of the lexicon were evaluated and confirmed after the presentation of the selected standard references and the intensities of each attribute were determined (4 h). In this session, panelists rated the intensities of samples using a 15-point scale (0 = none; 15 = extreme). The attribute intensities were assessed using this 0–15 point scale, drawing on prior sensory studies [14,15,21,22,23,24]. If differences were detected in the panel results, consensus on the ratings was reached through open discussion and a supplementary test. Another test was conducted to minimize rating discrepancies among the panelists using standard references (2 h).

In the concluding session (lasting 6 h), panelists assessed the selected standard samples according to the final testing procedure. Subsequently, they examined attributes of mushroom samples and their scores were evaluated for consistency. Each sample received intensity ratings in triplicate during this final session. Following the Latin square serving order, the presentation order of the samples was randomized using three-digit numbers [25,26]. To cleanse any residual taste or mushroom remnants between samples, panelists were provided with drinking water during each sample. A total of four mushroom samples were assessed in a randomized sequence, with three repetitions. The concluding session spanned 2 h over a period of 3 days.

### 2.4. Development of a Lexicon for Yellow Oyster Mushroom

Table 2 delineates the sensory attributes, definitions, and references for the samples examined in this research. To characterize appearance, odor/aroma, flavor/taste, and texture/mouthfeel, panelists devised a lexicon consisting of 20 attributes. For appearance, the descriptors (gray, yellow, and white) were selected. Odor/aroma attributes included fresh mushrooms and cooked mushrooms, reflecting their distinct aromas. The fresh mushroom odor encompassed scents such as oyster mushroom, woody, fishy, and seafood, whereas the cooked mushrooms aroma comprised mushroom, umami, and savory scents. Flavor/taste descriptors were finalized as sweet, salty, umami, and savory. Texture/mouthfeel was characterized by attributes such as chewiness, smooth texture, hardness, squishy texture, and slippery texture.

### 2.5. Consumer Acceptance Test

Consumer subjects (*n* = 100; females: 60, males: 40, age range: 20~58 years) were recruited to assess the acceptability of yellow oyster mushroom. It was not necessary for the subjects to have prior experience in sensory testing, but individuals who did not have an aversion to mushrooms were chosen. Each sample was presented to the consumers in two forms (raw and cooked) employing the method used in the descriptive analysis. Eight samples were randomly presented to the consumers as per the design of a randomized complete block [27,28]. The evaluation focused on various criteria such as overall_liking, appearance, overall_taste, sweetness, texture, savory flavor, MSG flavor, smell, color, purchasing intention, and recommendation. Water was provided between the samples to minimize the carryover taste from the previous sample. Consumers rated their liking on a nine-point hedonic scale ranging from 1(dislike extremely dislike to 9: extremely like) for nine attributes.

### 2.6. Statistical Analysis

Descriptive analysis was applied to compute the mean data values. One-way analysis of variance (ANOVA) was employed to examine the data related to attribute intensities and consumer acceptability to compare different samples. The criterion for determining a significant difference was set at *p* < 0.05. Duncan’s multiple range comparison was conducted as a post hoc test when a significant difference was observed with (α = 0.05). Principal component analysis (PCA) was used to elucidate the relationship between the samples and their sensory attributes, while partial least square regression (PLSR) analysis was employed to explore the associations among samples, descriptive attributes, and consumer acceptability. All statistical analyses were executed using IBM SPSS (Statistical Package for Social Science, ver. 25.0, Chicago, IL, USA) and the XLSTAT trial version 2024.2 (Addinsoft, Paris, France).

## 3. Results and Discussion

### 3.1. Intensites of Sensory Attributes from Descriptive Analysis

The intensities of sensory attributes for four types of oyster mushrooms are detailed in Table 3. The panelists identified 20 attributes for the four oyster mushroom samples. Among these, six attributes related to savory or umami-like odors and tastes included seafood odor (fresh), fishy odor (fresh), umami odor (cooked), nutty odor (cooked), umami taste, and MSG taste. The intensities of sensory attributes were rated using a 0–15 point scale, ranging from 0 (none) to 15 (extremely strong) based on previous sensory studies [14,15,21,22,23,24]. Chun et al. [14] introduced a descriptive sensory flavor lexicon for mushrooms, developing 27 flavor attributes that were categorized into two groups: musty and non-musty. These attributes included umami-like taste and odors, like fishy, shellfish, umami, and vegetable protein. The sensory study on shiitake mushrooms outlined descriptive attributes across three appearance attributes, six flavor attributes (mushroom flavor, fresh shiitake, rancid, nutty, roasted, bread), and two texture attributes (hardness, sponginess) [29]. Pinho et al. [30] developed six aroma attributes of wild mushrooms - mushroom-like, farm-feed, floral, honeylike, hay-like, and nutty- and investigated the relationship between sensory attributes and volatiles.

Our results show that, except for appearance, seven attributes—fresh mushroom smell, seafood smell (fresh mushroom), fishy smell (fresh mushroom), umami smell (cooked mushroom), nutty smell (cooked mushroom), salty taste, and MSG taste—exhibited significant differences among samples (*p* < 0.001). Sample 483 displayed the highest intensity scores for sensory attributes including seafood smell (fresh mushroom), fishy smell (fresh mushroom), nutty smell (cooked mushroom), and MSG taste. Mushrooms are increasingly gaining attention as future food ingredients due to their rich nutrition and distinctive taste and aroma. Umami, the quintessential taste of mushrooms, is characterized by a meaty, brothy, or savory flavor resulting from free amino acids, specifically L-glutamic acid and, L-aspartic acid, and 5′-nucleotides found in mushrooms, meat, fish, seafood, and vegetables [31]. The yellow oyster mushroom samples (483, 629, and 745) exhibited higher intensities of seafood smell (fresh mushroom), fishy smell (fresh mushroom), as well as umami smell (cooked mushroom), nutty smell (cooked mushroom), and umami taste, compared to the typical oyster mushroom sample (548). Our results indicate that the cooked yellow oyster mushroom presents a pleasant savory aroma, such as umami and nutty, whereas the fresh variety is characterized by seafood and fishy odor. Rotola-Pukkila et al. [32] noted that free amino acids and 5′-nucleotides, which are components of umami, were detected in cooked samples, but not in fresh samples. They found that thermal cooking of mushrooms leads to the release of taste and flavor compounds, although the specific compounds released vary depending on the type of mushroom and the cooking temperature used.

### 3.2. Principal Component Analysis (PCA)

**Figure 2:**
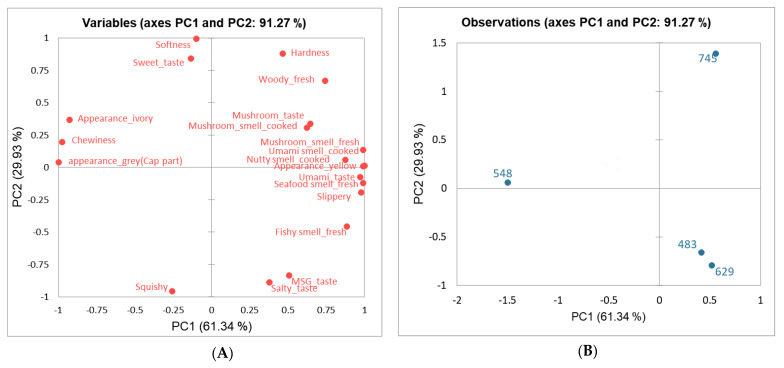
PC loadings regarding scores of the sensory attributes (**A**) and yellow oyster mushroom (**B**) evaluated by panels.

Figure 2 illustrates the visual image of all descriptive attributes and elucidates the relationship between sensory attributes and the samples using principal component analysis (PCA). The PCA biplot revealed 91.27% of the total variance, with Axes PC 1 (61.34%) and PC 2 (29.93%). Sensory attributes that contributed most to the right side of PC 1 (i.e., *X*-axis) included mushroom taste and smell, umami smell and taste, nutty smell, seafood smell, and slippery mouthfeel. Descriptors located to the left side of PC 1 encompassed chewiness and appearance, with sample 548 associated with these descriptors. Attributes on the positive side of PC 2 (i.e., *Y*-axis) included softness, hardness (fresh), and sweet taste, and with sample 745 located here. Conversely, attributes such as squishy texture, salty taste, MSG taste, and fishy smell (fresh) were found on the negative side of PC 2, with samples 483 and 629 located on this side.

### 3.3. Consumer Acceptability

Table 4 presents the results of the consumer acceptability test for oyster mushrooms. A total of 100 consumer panels tested the samples and rated each for appearance, smell, savory flavor, sweetness, texture/mouthfeel, overall liking, overall taste, inclination to try again (purchase intention), and recommendation intention. All attributes of the samples exhibited significant differences (*p* < 0.05), with the sample 548, a general oyster mushroom, achieving the highest acceptability scores among all samples. This sample is considered a traditional oyster mushroom, which is thought to be preferred due to its familiarity to consumers and its long-standing presence in the market. Conversely, yellow oyster mushrooms are perceived as less acceptable because they are less familiar and notcommonly consumed in our food culture. Consumers also assessed overall sensory intensity and differentiated overall acceptance [2]. Furthermore, there is no significant difference between the yellow oyster mushrooms.

### 3.4. Correlations between Descriptive Attributes and Consumer Acceptability

**Figure 3:**
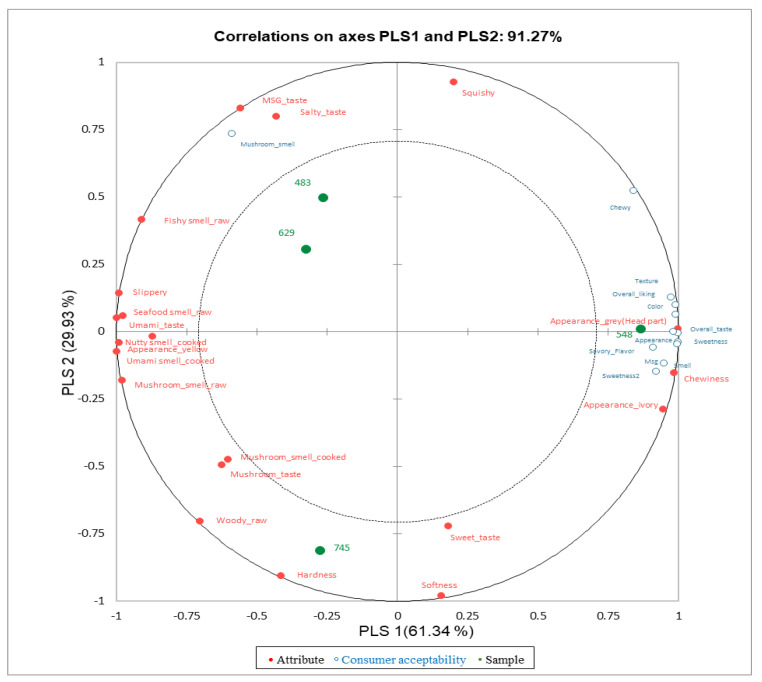
PLSR result indicating the association between sensory attributes, consumer acceptability, and yellow oyster mushroom samples.

Figure 3 displays the correlations between sensory attributes, consumer acceptability, and oyster mushroom samples. Partial least square regression (PLSR) analysis was employed to examine the correlations between descriptive attributes and consumer acceptability of the samples. PLSR analysis has proven to be an effective statistical method for analyzing and visually representing the correlation between two sets of data [33,34]. The analysis indicated that a total of 85.9% of the variance is explained by PLS 1 (61.34%) and PLS 2 (29.93%). The sample 548 (general oyster mushroom), which received the highest score for consumer acceptability, was situated on the right side of PLS 1 and was significantly associated with attributes of consumer acceptability. This sample was closely linked to various attributes, including overall liking, appearance, overall taste, savory flavor, sweetness, MSG flavor, color, texture, and smell. This result has some limitations because, although descriptive analysis by trained panels provides more specific and detailed information, evaluation from consumers tend to focus on overall differences and preferences [2,35]. Cho et al. [36] reported on the aroma characteristics of pine mushrooms using both gas chromatography–olfactometry and sensory analysis, finding that attributes such as piney, meaty, and floral were closely correlated. In the descriptive analysis, yellow oyster mushrooms demonstrated a greater affinity for umami-related tastes and flavors, including fishy smell, seafood smell, and MSG taste. The sample 745, positioned on the negative side of PLS 2, was strongly associated with attributes of hardness, softness (texture), sweet taste, mushroom smell, and mushroom taste. Conversely, the 483 and 629, located on the positive side of PLS 2, were associated with MSG taste, salty taste, squishy texture, and fishy smell (fresh mushroom).

## 4. Conclusions

This study identified the descriptive sensory characteristics and consumer acceptability of one general oyster mushroom and three selectively bred yellow oyster mushrooms. To describe characteristic appearances, 20 attributes were developed, covering colors (grey, yellow, and white), odors (fresh: mushroom, woody, fishy, and seafoody; cooked: mushroom, umami, and savory), flavors/tastes (sweet, salty, umami, and savory), and textures/mouthfeel (chewiness, smoothness, hardness, squishiness, and slipperiness). Consumer tests involving 100 participants revealed that the general oyster mushroom attained the highest acceptability scores among all tested samples. The other yellow oysters did not display significant differences in terms of sample acceptability. Sample 548 (general oyster mushroom) exhibited a strong correlation with all consumer acceptability attributes. 745 was closely associated with attributes of hardness, softness (texture), sweet flavor, mushroom smell, and mushroom taste. The 483 and 629 correlated with MSG taste, salty taste, squishy texture, and fishy smell (fresh mushroom). Yellow oyster mushrooms are g arnering significant interest as potential functional materials due to their demonstrated anti-cancer, anti-oxidant, and hyperlipidemic properties [18]. Additionally, there is a growing need to develop and cultivate mushrooms of various colors, such as yellow and pink, to satisfy consumer demand for color-rich foods. These newly developed superior mushroom lines could serve as a basis for functional foods, cosmetics, and healthy food products. These insights can guide the development of new cultivars that enhance the taste and texture of yellow oyster mushrooms and support the development of mushroom-based products to boost consumption.

## Figures and Tables

**Table 1:** Sample information.

Mushroom Strain	Information	Random Sampling Number
Pc-21-ja196 (KMCC0422 × KMCC2159)	New strain of yellow oyster mushroom (*Pleurotus citrinopileatus*) from two parent strains (KMCC0422 and KMCC2159) developed by National Institute of Horticultural and Herbal Science in 2021	483
Otari (KMCC5272)	Oyster mushroom (*Pleurotus ostreatus*) cultivar, ‘Otari’, developed by National Institute of Horticultural and Herbal Science in 2021	548
Pc-21-ba276 (KMCC0429 × KMCC2159)	New strain of yellow oyster mushroom (*Pleurotus citrinopileatus*) from two parent strains (KMCC0429 and KMCC2159) developed by National Institute of Horticultural and Herbal Science in 2021	629
Jangdari (KMCC0422)	Yellow oyster mushroom (*Pleurotus citrinopileatus)* cultivar, ‘Jangdari’, developed by National Institute of Horticultural and Herbal Science in 2012	745

**Table 2:** Sensory attributes, definitions, and physical standards of yellow oyster mushroom.

Attributes (Descriptor)	Definition	Reference *
Appearance		
Gray	Intensity of gray color	Jongienara Co.(Yangju, Republic of Korea) 200 colors, No. 103Y/DK (10) ^(1)^
Yellow	Intensity of yellow color	Jongienara Co. (Yangju, Republic of Korea) 200 colors, No. 13Y/S (9)
White	Intensity of white color	Jongienara Co. (Yangju, Republic of Korea) 200 colors, No. 112N9 (8)
Odor/Aroma(smell)		
Fresh mushroom		
Oyster mushroom smell	The smell associated with raw oyster mushroom	Commercial oyster mushroom (Iksan, Jeollabuk do, Republic of Korea) (9)
Woody smell	The woody smell associated with pine bark	Pine bark (5)
Fishy smell	The fishy smell associated with scallop boiled in water	Scallop in boiled water (scallops 70 g + water 630 mL boiled for 10 min) (9)
Seafood smell	The smell intensity associated with seafood	Crab boiled in water (crab 300 g + water 1000 mL boiled for 5 min) (10)
Cooked mushroom		
Mushroom smell	The smell associated with cooked mushroom	Cooked oyster mushroom (Iksan, Jeollabuk do, Republic of Korea) (8)
Umami odor	The umami odor associated with crab boiled in water	Crab boiled in water (crab 300 g + water 1000 mL boiled for 5 min) (10)
Savory odor	Savory odor, intensity of corn tea	Corn tea (corn 10 g + water 830 mL boiled for 10 min) (8)
Flavor/Taste		
Mushroom flavor & taste	The flavor and taste when chewing the mushroom	Cooked oyster mushroom (Iksan, Jeollabuk-do, Republic of Korea) (8)
Sweet taste	Typical taste of sucrose	(Strong) Sugar 1% + Water 99% (15) (Weak) Sugar 0.1% + Water 99.9% (1)
Salty taste	Typical taste of salt	(Strong) Salt 0.1% + Water 99.9% (13) (Weak) Salt 0.05% + Water 99.95% (1)
Umami taste	Typical taste of MSG (Monosodium glutamate)	(Strong) MSG (Miwon, Daesang Co., Yongin, Gyeonggi-do, Republic of Korea) 0.1% + Water 99.9% (15) (Normal) MSG (Miwon, Daesang Co., Yongin, Gyeonggi-do, Republic of Korea) 0.05% + Water 99.95% (8) (Weak) MSG (Miwon, Daesang Co., Yongin, Gyeonggi-do, Republic of Korea) 0.01% + Water 99.99% (1)
Savory flavor & taste	Typical savory taste	Crab boiled in water (crab 300 g + water 1000 mL boiled for 5 min) (10)
Texture/Mouthfeel		
Chewiness	Degree to which there is a mouthfeel sensation of labored chewing due to sustained, elastic resistance from jelly	Jelly Straws (Tsang Lin Industries Corp., Taichung, Taiwan) (12)
Smooth texture	Soft feel in the mouth (degree of smooth texture associated with milk pudding)	Milk pudding (Peacock, Emart, Jinchon, Chungcheongbuk-do, Republic of Korea) (13)
Hardness	Degree of the quality or state of being hard	Raw eggplant (9)
Squishy texture	Degree of being soft and wet	Eggplant steamed for 5 min (12)
Slippery texture	Degree to which there is a smooth and wet surface on the mushroom (degree of slipperiness)	Canned ecliptic peaches (Ottogi, Goseong, Gyeongnam, Republic of Korea) (13)

* References were prepared 24 h in advance, stored in a refrigerator, and retrieved one hour prior the testing session. ^(1)^ This number represents intensity of the standard reference utilizing the same 15-point scale (slight: 1, moderate: 5–9, strong: 10–15).

**Table 3:** Intensities of sensory attributes of yellow oyster mushroom.

	Mushroom Samples				*p*-Value ^(1)^
	483	548	629	745	
	Mean SD	Mean SD	Mean SD	Mean SD	
Appearance_grey (Cap part)	1.00 ± 0.00 ^b^	9.17 ± 1.07 ^a^	1.00 ± 0.00 ^b^	1.00 ± 0.00 ^b^	<0.001
Appearance_ivory (Body part)	8.67 ± 1.41	10.07 ± 2.50	8.40 ± 1.74	9.03 ± 1.33	0.197
Appearance_yellow (Cap part)	9.43 ± 0.90 ^a^	1.10 ± 0.22 ^b^	8.50 ± 1.45 ^a^	9.33 ± 0.93 ^a^	<0.001
Mushroom smell_fresh	10.40 ± 1.40 ^a^	8.80 ± 0.45 ^b^	10.37 ± 0.78 ^a^	10.70 ± 0.88 ^a^	<0.001
Seafood smell_fresh	9.43 ± 1.16 ^a^	6.40 ± 1.26 ^b^	8.83 ± 1.23 ^a^	9.00 ± 1.15 ^a^	<0.001
Woody_fresh	4.13 ± 1.23	3.70 ± 1.32	4.17 ± 1.53	4.87 ± 1.25	0.289
Fishy smell_fresh	8.37 ± 1.05 ^a^	4.80 ± 1.43 ^c^	8.20 ± 1.16 ^a^	6.80 ± 1.58 ^b^	<0.001
Mushroom smell_cooked	9.57 ± 1.01	9.57 ± 1.98	10.17 ± 1.27	10.13 ± 1.22	0.641
Umami smell_cooked	9.50 ± 1.03 ^a^	7.10 ± 1.43 ^b^	9.67 ± 1.49 ^a^	9.73 ± 1.30 ^a^	<0.001
Nutty smell_cooked	8.63 ± 1.30 ^a^	6.33 ± 1.56 ^b^	7.53 ± 1.32 ^ab^	8.23 ± 1.52 ^a^	<0.005
Mushroom taste	8.30 ± 1.14	8.27 ± 0.64	9.07 ± 1.64	9.07 ± 0.84	0.205
Sweet taste	6.30 ± 1.74	6.37 ± 1.46	5.70 ± 1.56	6.73 ± 2.10	0.613
Salty taste	7.83 ± 1.58 ^ab^	6.50 ± 1.65 ^bc^	8.90 ± 2.07 ^a^	5.97 ± 1.52 ^c^	<0.005
Umami taste	9.90 ± 1.32	8.27 ± 1.78	10.10 ± 1.74	9.87 ± 1.93	0.076
MSG taste	6.90 ± 1.98 ^a^	4.97 ± 1.69 ^b^	6.53 ± 1.42 ^a^	4.90 ± 1.36 ^b^	<0.005
Chewiness	9.53 ± 1.76	10.07 ± 1.64	9.57 ± 1.74	9.63 ± 1.63	0.885
Softness	7.20 ± 1.46	7.43 ± 1.38	7.20 ± 1.52	7.70 ± 1.54	0.853
Hardness	7.07 ± 1.39	7.03 ± 1.50	7.10 ± 1.16	7.53 ± 1.04	0.801
Squishiness	11.23 ± 1.13	11.23 ± 1.08	11.27 ± 1.24	11.10 ± 0.88	0.986
Slipperiness	10.87 ± 1.24	10.77 ± 1.32	10.87 ± 1.51	10.23 ± 1.16	0.662

^(1)^ *p*-value by ANOVA. ^a–c^ Different superscript letters indicate significant differences among groups at the α = 0.05 level, as determined Duncan’s multiple range test.

**Table 4:** Consumer acceptability of yellow oyster mushroom.

	Mushroom Samples				*p*-Value ^(1)^
	483	548	629	745	
	Mean SD	Mean SD	Mean SD	Mean SD	
Overall_liking	5.01 ± 1.89 ^b^	6.30 ± 1.93 ^a^	4.73 ± 2.03 ^b^	4.76 ± 1.92 ^b^	<0.001
Appearance	5.05 ± 1.75 ^b^	6.73 ± 1.86 ^a^	4.79 ± 2.04 ^b^	4.98 ± 1.77 ^b^	<0.001
Overall_taste	5.16 ± 1.96 ^b^	6.37 ± 2.08 ^a^	4.75 ± 2.01 ^b^	5.02 ± 1.90 ^b^	<0.001
Sweetness	4.96 ± 1.59 ^b^	6.25 ± 1.64 ^a^	5.00 ± 1.82 ^b^	5.04 ± 1.60 ^b^	<0.001
Texture	5.46 ± 1.77 ^b^	6.41 ± 1.91 ^a^	5.10 ± 1.96 ^b^	5.17 ± 1.76 ^b^	<0.001
Savory Flavor	5.54 ± 1.73 ^b^	6.20 ± 1.73 ^a^	5.01 ± 1.80 ^c^	5.41 ± 1.47 ^bc^	<0.001
MSG flavor	4.95 ± 1.55 ^b^	5.54 ± 1.53 ^a^	4.85 ± 1.62 ^b^	4.95 ± 1.21 ^b^	0.014
Smell	4.68 ± 2.10 ^b^	5.93 ± 1.88 ^a^	5.03 ± 1.88 ^b^	4.96 ± 1.93 ^b^	<0.001
Color	5.13 ± 1.93 ^b^	6.41 ± 2.09 ^a^	5.24 ± 1.85 ^b^	5.10 ± 1.88 ^b^	<0.001
Purchase	4.15 ± 1.99 ^b^	5.93 ± 2.52 ^a^	4.20 ± 2.14 ^b^	4.24 ± 2.19 ^b^	<0.001
Recommend	4.42 ± 1.94 ^b^	5.99 ± 2.51 ^a^	4.36 ± 2.08 ^b^	4.32 ± 2.08 ^b^	<0.001

^(1)^ *p*-value by ANOVA. ^a–c^ Different superscript letters mean significantly different between groups at α = 0.05 level with Duncan’s multiple range test.

## Data Availability

The original contributions presented in the study are included in the article, further inquiries can be directed to the corresponding author.

## References

[1] Devi P.V., Islam J., Narzary P., Sharma D., Sultan F. (2024). Bioactive compounds, nutraceutical values and its application in food product development of oyster mushroom. J. Future Foods.

[2] Aisala H., Laaksonen O., Manninen H., Raittola A., Hopia A., Sandell M. (2018). Sensory properties of Nordic edible mushrooms. Food Res. Int..

[3] Wan Mahari W.A., Peng W., Nam W.L., Yang H., Lee X.Y., Lee Y.K., Liew R.K., Ma N.L., Mohammad A., Sonne C. (2020). A review on valorization of oyster mushroom and waste generated in the mushroom cultivation industry. J. Hazard. Mater..

[4] Zhou T., Hu W., Yang Z., Li J., Zeng X. (2023). Study on nutrients, non-volatile compounds, volatile compounds and antioxidant capacity of oyster mushroom cultivated with corn distillers’ grains. LWT.

[5] Mishra V., Tomar S., Yadav P., Singh M.P. (2021). Promising anticancer activity of polysaccharides and other macromolecules derived from oyster mushroom (*Pleurotus* sp.): An updated review. Int. J. Biol. Macromol..

[6] Himani K., Vani G., Vandana G., Supriya M., Sameeksha M. (2020). Hypoglycemic and hyperlipidemic effect of oyster mushroom (*Pleurotus ostreatus*) extract in Indian obese children. Genes Dis..

[7] Stastny J., Marsik P., Tauchen J., Bozik M., Mascellani A., Havlik J., Landa P., Jablonsky I., Treml J., Herczogova P. (2022). Antioxidant and anti-inflammatory activity of five medicinal mushrooms of the genus Pleurotus. Antioxidants.

[8] Wasser S.P. (2002). Medicinal mushrooms as a source of antitumor and immunomodulating polysaccharides (minireview). Appl. Microbiol. Biotechnol..

[9] Lindequist U., Niedermeyer T.H.J., Julich W.D. (2005). The pharmacological potential of mushrooms: Evidence-based complement. Altern. Med..

[10] Kortei N.K., Odamtten G.T., Obodai M., Appiah V., Akuamoa F., Adu-Bobi A.K., Annan S.N.Y., Armah J.O., Acquah S.A. (2014). Evaluating the effect of gamma radiation on the total phenolic content, flavonoids and antioxidant activity of dried *P. ostreatus* (Jacq.ex.Fr.) Kummer stored in packaging materials. Adv. Pharm..

[11] Pachekrepapol U., Thangrattana M., Kitikangsadan A. (2022). Impact of oyster mushroom (*Pleurotus ostreatus*) on chemical, physical, microbiological and sensory characteristics of fish burger prepared from salmon and striped catfish filleting by-product. Int. J. Gastron. Food Sci..

[12] Luo Q., Jiang C., Yan Y., Li C., Fang Z., Hu B., Wang C., Chen S., Wu W., Li X. (2022). Effect of different cooking methods on the nutrients, antioxidant and hypoglycemic activities of *Pleurotus cornucopiae* in vitro simulated digestion. Food Res. Int..

[13] Li X., Chen G., Ezemaduka A.N., Luo N., Yu H., Wang M. (2023). The yields and quality of golden oyster mushroom cultivated on common reed substrates. J. Food Compos. Anal..

[14] Chun S., Chambers E., Han I. (2020). Development of a sensory flavor lexicon for mushrooms and subsequent characterization of fresh and dried mushrooms. Foods.

[15] Guinard J.X., Myrdal Miller A., Mills K., Wong T., Lee S.M., Sirimuangmoon C., Schaefer S.E., Drescher G. (2016). Consumer acceptance of dishes in which beef has been partially substituted with mushrooms and sodium has been reduced. Appetite.

[16] Chun S., Chambers E., Chambers D. (2005). Perception of pork patties with shiitake (*Lentinus edode* P.) mushroom powder and sodium tripolyphosphate as measured by Korean and United States consumers. J. Sens. Stud..

[17] Mohajan S., Orchy T.N., Farzana T. (2018). Effect of incorporation of soy flour on functional, nutritional, and sensory properties of mushroom–moringa-supplemented healthy soup. Food Sci. Nutr..

[18] Oh M., Lim J., Oh Y., Shin P., Jang K., Kong W., Yoo Y. (2017). Characteristics and breeding of a cultivar *Pleurotus citrinopileatus* ‘Jangdari’. J. Mushrooms.

[19] MacFie H., Bratchell N., Greenhoff K., Vallis L.V. (1989). Designs to balance the effect of order of presentation and first-order carry-over effects in hall tests. J. Sens. Stud..

[20] Lawless H.T., Heymann H. (2010). Sensory Evaluation of Food: Principles and Practices.

[21] Hongsoongnern P., Chambers E. (2008). A lexicon for texture and flavor characteristics of fresh and processed tomatoes. J. Sens. Stud..

[22] Suwonsichon S., Chambers E., Kongpensook V., Oupadissakoon C. (2012). Sensory lexicon for mango as affected by cultivars and stages of ripeness. J. Sens. Stud..

[23] Oliver P., Cicerale S., Pang E., Keast R. (2018). Developing a strawberry lexicon to describe cultivars at two maturation stages. J. Sens. Stud..

[24] Wu G., Ross C.F., Morris C.F., Murphy K.M. (2017). Lexicon Development, Consumer Acceptance, and Drivers of Liking of Quinoa Varieties. J. Food Sci..

[25] Hwang S.H., Hong J.H. (2015). Determining the most influential sensory attributes of nuttiness in soymilk: A trial with Korean consumers using model soymilk. J. Sens. Stud..

[26] Schlich P. (1993). Uses of change-over designs and repeated measurements in sensory and consumer studies. Food Qual. Prefer..

[27] Kwon Y.S., Ju S. (2018). Sensory evaluation of commercial ready-to-eat rice between trained panelist and consumer. British Food J..

[28] Gwak M.J., Chung S.J., Kim Y. (2012). Sensory drivers of liking for Adlay (Coix lacryma-jobi) tea. Korean J. Food Cult..

[29] Politowicz J., Lech K., Lipan L., Figiel A., Carbonell-Barrachin Á.A. (2018). Volatile composition and sensory profile of shiitake mushrooms as affected by drying method. J. Sci. Food Agric..

[30] Pinho P.G., Ribeiro B., Gonçalves R.F., Baptista P., Valentão P., Seabra R.M., Andrade P.B. (2008). Correlation between the Pattern Volatiles and the Overall Aroma of Wild Edible Mushrooms. J. Agric. Food Chem..

[31] Manninen H., Rotola-Pukkila M., Aisala H., Hopia A., Laaksonen T. (2018). Free amino acids and 5′-nucleotides in Finnish forest mushrooms. Food Chem..

[32] Rotola-Pukkila M., Yang B., Hopia A. (2019). The effect of cooking on umami compounds in wild and cultivated mushrooms. Food Chem..

[33] Chung S.J., Heymann H., Gruen I.U. (2003). Application of PGPA and PLSR in correlating sensory and chemical data sets. Food Qual. Prefer..

[34] Chung L., Chung S.J. (2008). Understanding the factors affecting the acceptance for fermented soybean products. Food Sci. Biotechnol..

[35] Ares G., Varela P. (2017). Trained vs. consumer panels for analytical testing: Fueling a long lasting debate in the field. Food Qual. Prefer..

[36] Cho I.H., Lee S.M., Kim S.Y., Choi H.-K., Kim K.-O., Kim Y.-S. (2007). Differentiation of aroma characteristics of pine-mushrooms (*Tricholoma matsutake* sing.) of different grades using gas chromatography−Olfactometry and sensory analysis. J. Agric. Food Chem..

